# Supplementary figures and images for: A zinc-finger fusion protein refines Gal4-defined neural circuits
Source: Mol Brain. 2018 Aug 20;11:46. doi: 10.1186/s13041-018-0390-7 (PMC6102859; doi:10.1186/s13041-018-0390-7)

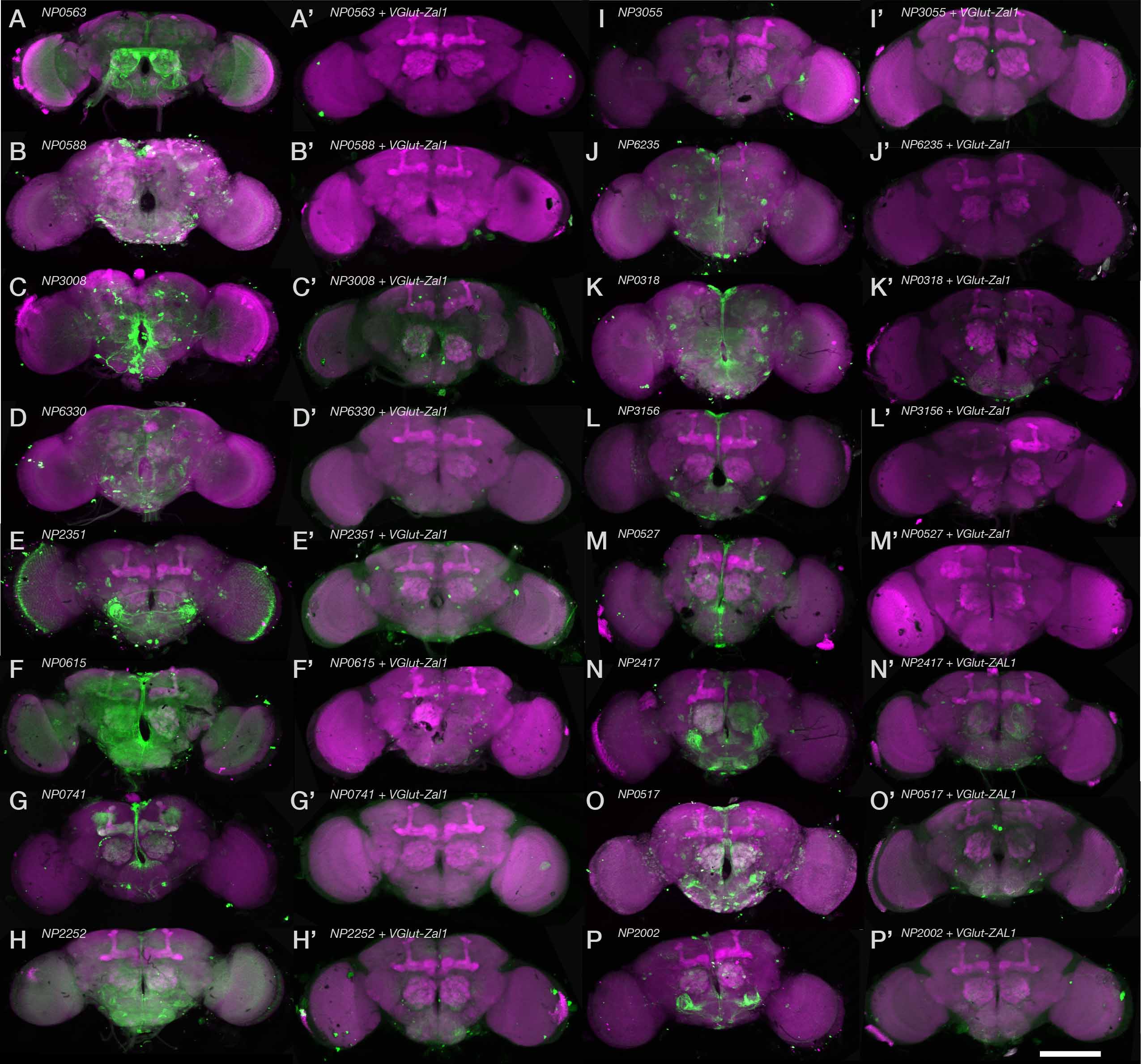

Supplement: Supplementary file 1 — Figure S1. Expression patterns of NP lines with and without VGlut-Zal1 AND operation. Expression patterns of 16 NP drivers in the adult brain, alone and in combination with VGlut-Zal1. All brains are stained with α-GFP (green) and α-DLG (magenta). A–R. Expression patterns of NP0563, NP0588, NP3008, NP6330, NP2351, NP0615, NP0741, NP2252, NP3055, NP6235, NP0318, NP3156, NP0527, NP2417, NP0517, and NP2002 Gal4 enhancer trap lines. A’–R’. The intersectional expression patterns in combinations with VGlut-Zal1 are shown in the panels on the right. Several brains lack appreciable α-GFP signal, including NP0563, NP0588, and NP0741. White scale bar represents 200 μm. (JPG 335 kb) [file 13041_2018_390_MOESM1_ESM.jpg]

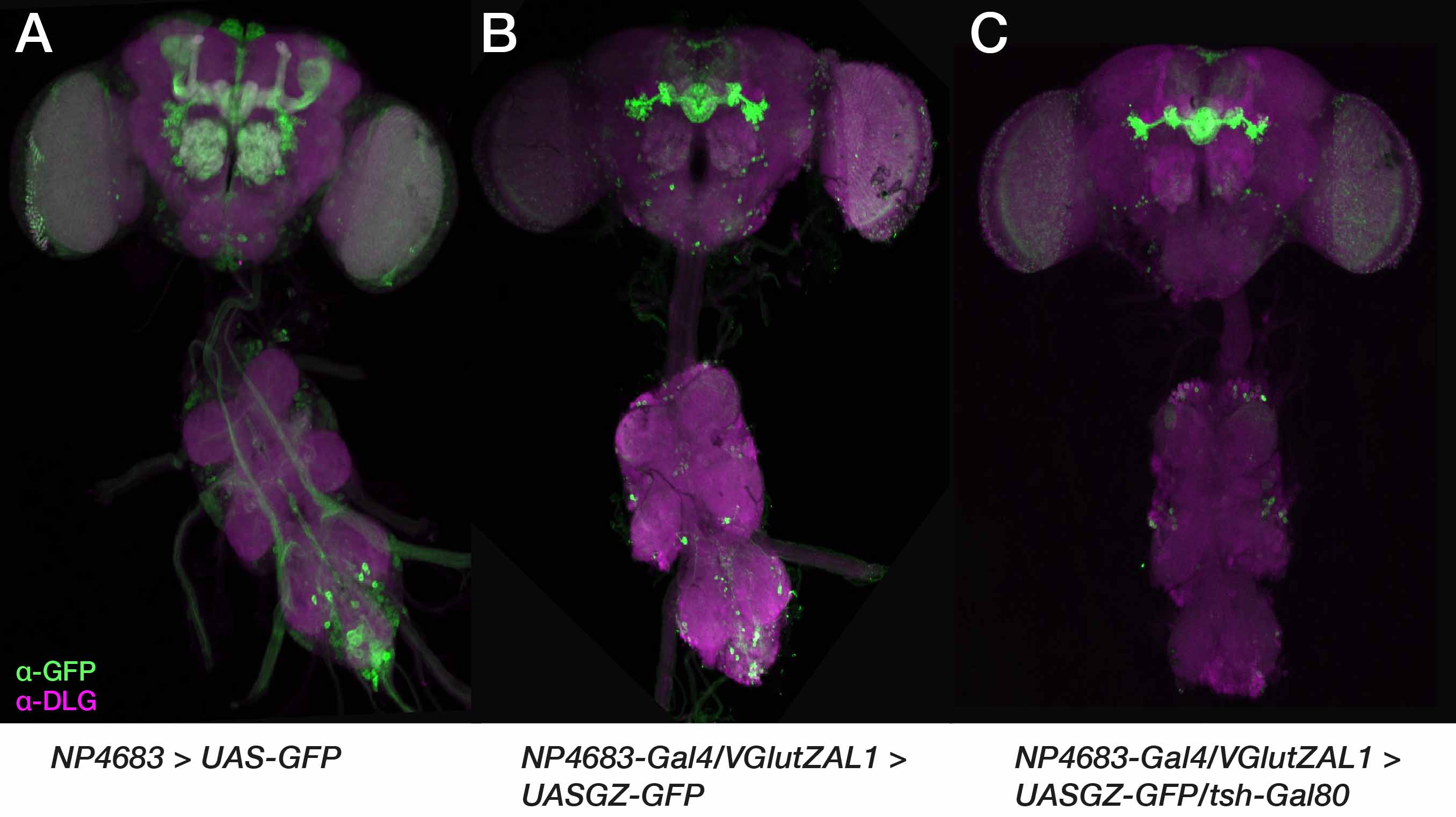

Supplement: Supplementary file 2 — Figure S2. Gal80 represses expression activated by the Gal4-Zal1 dimer. A. The enhancer trap line NP4683 expressed in a wide range of brain cells, as indicated by α-GFP (green) immunostain. The neuropils are stained with α-DLG (magenta). B. Intersection with VGlut-Zal1 reduced the expression range, though left expression in several areas including the ellipsoid body, subesophageal zone, and the ventral nerve cord. C. Combination with tsh-Gal80 left the ellipsoid body brightly stained, while reducing expression in the subesophageal zone, and the ventral nerve cord. (JPG 170 kb) [file 13041_2018_390_MOESM2_ESM.jpg]
